# Degradable Polymer-Based Oil–Water Separation Materials Prepared by High Internal Phase Emulsion Templating Method and Silica-Modification

**DOI:** 10.3390/polym17243254

**Published:** 2025-12-06

**Authors:** Yunpeng Hu, Jianqiao Lu, Maoning Li, Qingyuan Du, Jing Zhao, Dandan Li, Xiangrui Meng, Yu Nan, Zhi Zhang, Dazhi Sun

**Affiliations:** 1 SUSTech-Nanke Materials Joint Laboratory for Innovative Materials, Department of Materials Science and Engineering, Southern University of Science and Technology, Shenzhen 518055, China; 12432475@mail.sustech.edu.cn (Y.H.); 12312703@mail.sustech.edu.cn (J.L.); 12531268@mail.sustech.edu.cn (M.L.); 12331179@mail.sustech.edu.cn (Q.D.); 12149013@mail.sustech.edu.cn (J.Z.); lidd@sustech.edu.cn (D.L.); mengxr12900@outlook.com (X.M.); 2 Nanke Materials Co., Ltd., Shenzhen 518000, China; nanyu@nanketech.com (Y.N.); zhangzhi@nanketech.com (Z.Z.)

**Keywords:** oil–water separation, high internal phase emulsion, degradable porous polymer, silica deposition, superhydrophobicity

## Abstract

The development of oil–water separation materials that combine high separation efficiency, robust mechanical properties, and environmental degradability remains a significant challenge. This study presents a novel degradable and superhydrophobic porous material fabricated via a multi-step process. A porous foam was first synthesized from degradable poly(ε-caprolactone-co-2-ethylhexyl acrylate) using a high internal phase emulsion templating technique. The foam was subsequently modified through in situ silica (SiO_2_) deposition via a sol–gel process, followed by grafting with hydrophobic hexadecyltrimethoxysilane (HDTMS) to produce the final oil–water separation porous materials. Various characterization results showed that the optimized material featured a hierarchical pore structure in micro scales and the porosity of the foam remained ~90% even after the 2-step modification. Mechanical tests indicate that the modified material exhibited significantly enhanced compressive strength and the water contact angle measurements revealed a superhydrophobic surface with a value of approximately 156°. The prepared material demonstrated excellent oil/water separation performance with notable absorption capacities ranging from 4.11 to 4.90 g/g for oils with different viscosity. Additionally, the porous material exhibited exceptional cyclic stability, maintaining over 90% absorption capacity after 10 absorption-desorption cycles. Moreover, the prepared material achieved a mass loss of approximately 30% within the first 3 days under alkaline hydrolysis conditions (pH 12, 25 °C), which further escalated to ~70% degradation within four weeks. The current work establishes a feasible strategy for developing sustainable, high-performance oil–water separation materials through rational structural design and surface engineering.

## 1. Introduction

The escalating global challenge of water pollution, driven by industrial discharges, petrochemical operations, and accidental oil spills, poses a severe threat to ecosystems and human health [1,2,3]. The contamination of water resources by oil and other pollutants has grown to alarming proportions, undermining the quality of water in both freshwater and marine environments. This not only affects aquatic life but also endangers human communities that depend on these water bodies for drinking, irrigation, and recreational activities [4]. Consequently, there has been an increasing demand for efficient and sustainable oil–water separation technologies capable of addressing this pervasive issue [5,6].

Conventional oil–water separation methods, including gravity separation, centrifugation, and membrane filtration, have been employed to mitigate this problem [7,8,9]. While these techniques are effective to some degree, they often suffer from significant limitations that hinder their broad applicability, particularly in decentralized settings or scenarios where low-cost, energy-efficient solutions are required. Gravity separation, for instance, relies on the density difference between oil and water to separate the two phases. This method can also be slow and inefficient when dealing with emulsified oil–water mixtures, which are more difficult to separate [7]. Centrifugation, while more efficient, requires significant energy input and specialized equipment, making it unsuitable for widespread use in low-resource settings [10]. Membrane filtration offers a promising alternative but is often plagued by issues such as fouling, clogging, and the need for frequent cleaning, all of which reduce the long-term effectiveness of the process [9,11].

After discussing conventional methods such as gravity separation, centrifugation, and membrane filtration, which have limitations in terms of efficiency and energy consumption, advanced material-based approaches have emerged as promising alternatives. Among these, High Internal Phase Emulsion (HIPE) templating has gained attention for its ability to create highly porous materials with interconnected pore structures [12,13]. In a HIPE, the dispersed phase constitutes more than 74% of the total volume, resulting in a foam with high porosity and a robust framework. This technique allows for precise control over the pore structure, making it ideal for applications like water evaporator [14] and oil–water separation [15].

In response to these challenges, advanced porous materials with engineered surface wettability have emerged as a promising frontier in the development of oil–water separation technologies [16,17]. By precisely tailoring the surface chemistry and constructing hierarchical microstructures, these materials can achieve selective permeability, allowing one phase (oil or water) to pass through while repelling the other [18,19]. In particular, porous materials featuring a two-level pore architecture—large primary pores interconnected by smaller window pores—have shown distinct advantages in liquid transport regulation. The large pores provide high storage capacity and rapid oil flow pathways, while the window pores ensure effective interconnectivity and contribute to selective penetration, thereby improving the overall separation efficiency. The development of such materials is based on the principle of surface wettability, which determines the interaction between a solid surface and a liquid. Among the various wettability paradigms, the creation of superhydrophobic and superhydrophilic surfaces has proven to be particularly effective for separating immiscible oil–water mixtures. Superhydrophobic surfaces repel water, while superhydrophilic surfaces attract oil, enabling highly efficient separation driven by capillary forces [20]. This approach leverages the inherent properties of the materials, facilitating the separation process without requiring external energy input.

The promising results achieved with these advanced materials have garnered significant attention. Materials fabricated from polytetrafluoroethylene (PTFE), polypropylene (PP), and polyvinylidene fluoride (PVDF) are widely used in oil–water separation due to their excellent chemical resistance and mechanical robustness. These properties make them ideal for harsh industrial environments where oil–water separation is required. For instance, Xu et al. [21] introduced a bioinspired multifunctional PVDF membrane, modified with MXene and silica nanoparticles, which achieved over 99% separation efficiency for oil–water emulsions. This material demonstrated excellent performance in removing complex pollutants from oily wastewater, maintaining high stability through five filtration cycles. Similarly, Yu et al. [22] modified PTFE membranes by grafting polydopamine (PDA) and glucosamine, which enhanced hydrophilicity (reducing the water contact angle from 123° to 13°) and antibacterial properties (99% inhibition of Staphylococcus aureus). The modified PTFE membranes achieved a separation efficiency of 97.5% for iso-paraffin emulsions, with a flux rate of 90 L m^−2^ h^−1^ bar^−1^.

However, despite these remarkable performance characteristics, the non-degradable nature of these polymers poses a significant environmental challenge. Once their service life ends, these materials contribute to the growing burden of plastic waste [23,24]. Over time, they can fragment into microplastics, which are notorious for their persistence in the environment and potential harm to aquatic life and human health [25,26]. This creates an inherent contradiction: while these materials address one environmental issue, they inadvertently exacerbate another.

To bridge this sustainability gap, researchers are increasingly focusing on developing high-performance separation materials fabricated from biodegradable substrates [27,28]. For example, Liu et al. [29]. fabricated superhydrophobic/superoleophilic bamboo cellulose foam using sol–gel and freeze-drying methods. This material demonstrated excellent oil absorption performance (ranging from 11.5–37.5 g/g) and high recyclability (31.5 g/g after 10 cycles), offering a more sustainable solution for oil–water separation. Mao et al. [30]. developed a starch-based superhydrophobic cryogel composite, P-CW@SC, through a two-step process involving carnauba wax and polydimethylsiloxane. This composite exhibited superhydrophobicity with contact angle of 154° and excellent oil absorption capacity up to 37.5 g/g, with over 95% oil–water separation efficiency, while remaining biodegradable.

In addition, aliphatic polyesters, particularly polycaprolactone (PCL), have emerged as promising candidates due to their biodegradability, biocompatibility, and tunable mechanical properties [31]. PCL undergoes hydrolytic degradation of its ester linkages under environmental conditions, breaking down into harmless products like water and carbon dioxide, making it a more environmentally friendly alternative to synthetic polymers [32]. However, porous foams based on neat PCL typically lack the optimal performance balance required for effective oil–water separation. While PCL is intrinsically hydrophobic, its inherent hydrophobicity remains insufficient to achieve the necessary level of water repellency [33,34]. More critically, the mechanical strength of neat PCL-based materials is often inadequate for continuous operation in demanding separation processes. Some researchers indicate that the incorporation of 2-ethylhexyl acrylate (EHA) as a comonomer significantly improves the mechanical resilience of the PCL matrix while preserving its inherent biodegradability [35,36].

To address the limitations of native PCL-based foams, this work proposes a HIPE-templated strategy for constructing degradable porous materials with tunable wettability and mechanical stability. In this approach, a vinyl-terminated PCL prepolymer is copolymerized with 2-ethylhexyl acrylate to generate a highly porous and interconnected foam suitable for oil–water separation. The foam is then further engineered through a two-step surface modification: silica deposition via a TEOS-based sol–gel process to introduce surface roughness, followed by hydrophobic grafting with hexadecyltrimethoxysilane (HDTMS) to lower the surface energy [37,38]. This combination of a degradable polymer matrix and controllable surface chemistry provides a feasible route toward environmentally friendly porous materials designed for selective oil uptake.

## 2. Materials and Methods

### 2.1. Materials

Polycaprolactone diol (PCL, number average molecular weight ~2000 g/mol), Span-80 (sorbitan oleate, USP), and acrylic acid were purchased from Aladdin Biochemical Technology Co., Ltd. (Shanghai, China). 4-Dimethylaminopyridine (C_7_H_10_N_2_, 99%) and calcium chloride (CaCl_2_, AR) were sourced from Shanghai Lingfeng Chemical Reagent Co., Ltd. (Shanghai, China). Dicyclohexylcarbodiimide (DCC), tetraethyl orthosilicate (TEOS), hexadecyltrimethoxysilane (HDTMS), 2-ethylhexyl acrylate (2-EHA, 99%), ethylene glycol dimethacrylate (EGDMA, 98%), potassium persulfate (KPS), and ammonium persulfate (APS), along with solvents such as toluene, acetone, anhydrous ethanol, and oxalic acid, were provided by Macklin Biochemical Technology Co., Ltd. (Shanghai, China).

The oil samples used in this study included n-hexane provided by Macklin Biochemical Technology, light fuel oil kerosene from Suzhou Shanjie New Material corporation, industrial lubricating oil from Kunlun Lubricant, and common edible oil from Arawana. This variety of oil types aims to provide a wide range of oil chemistries and viscosities.

### 2.2. Synthesis of Vinyl-Terminated PCL Prepolymer

A vinyl-terminated polycaprolactone (PCL) prepolymer was synthesized via an esterification reaction.

In a 500 mL round-bottom flask, polycaprolactone diol (44.30 g, ~22.15 mmol), acrylic acid (2.88 g, 40.00 mmol), and 4-dimethylaminopyridine (1.22 g, 10.00 mmol) were dissolved in 170 mL acetone, and the mixture was stirred continuously at room temperature until a homogeneous transparent solution was formed. Subsequently, dicyclohexylcarbodiimide (8.24 g, 40.00 mmol) was dissolved in 30 mL acetone and slowly added dropwise to the reaction mixture via a pressure-equalizing funnel. After complete addition, the reaction mixture was stirred at room temperature for 12 h.

Upon completion of the reaction, 0.1 g of deionized water was added to quench the reaction, and stirring was continued for an additional 2 h. The reaction mixture was then subjected to centrifugation to separate the upper clear liquid, which was subsequently poured into a large volume of deionized water to precipitate the product. This dissolution-precipitation purification process was repeated three times to thoroughly remove byproducts. The final white solid product was dried in a vacuum oven until constant weight, sealed, and stored at 4 °C for further use.

### 2.3. Fabrication of Porous Polymer Foams via High Internal Phase Emulsion Templating

Porous polymer foams were fabricated using the high internal phase emulsion (HIPE) templating method.

Water phase preparation: Potassium persulfate (0.21 g) and calcium chloride (3.654 g) were dissolved in 91.31 g of deionized water and stirred until homogeneous.

Oil phase preparation: The synthesized vinyl-terminated PCL prepolymer (2.1 g), toluene (2.1 g), Span-80 (2.1 g), 2-ethylhexyl acrylate (3.5 g), and ethylene glycol dimethacrylate (2.1 g) were mixed and stirred thoroughly.

The prepared oil phase was then transferred into a foaming machine container. Under continuous stirring, the water phase was slowly added at a rate of 30 mL/min. After the addition was complete, the mixture was stirred at high speed for an additional 8 min to form a stable high internal phase emulsion. The resulting emulsion was then quickly transferred to a specific mold and subjected to thermal polymerization at 60 °C for 24 h to solidify the foam. After polymerization, the foam was removed from the mold, washed with 75% (*v*/*v*) ethanol solution (3 × 40 mL) to remove unreacted monomers and emulsifiers, and finally dried at 40 °C.

The resulting pristine porous foam was primarily composed of poly(ε-caprolactone-co-2-ethylhexyl acrylate), which was designated as PCE.

### 2.4. Deposition of Tetraethyl Orthosilicate (TEOS)

Silica deposition was carried out on the polymer foam skeleton surface via a sol–gel process.

The prepared PCE foam samples were cut into uniform-sized specimens and randomly divided into six groups, with one group as a blank control (without TEOS deposition).

The deposition solution was prepared by mixing deionized water (12 g), concentrated hydrochloric acid (0.2 g, as a catalyst), tetraethyl orthosilicate (30 g), and anhydrous ethanol (80 g) and stirring the mixture. The other five groups of foam samples were completely immersed in the freshly prepared deposition solution and removed after 20, 40, 60, 80, and 100 min of immersion, respectively, to investigate the effect of deposition time on the coating. After removal, the samples were dried and cured at 60 °C in an oven.

The silica-deposited foams are denoted as x-PCE, where x represents the TEOS deposition time in minutes (e.g., 20-PCE, 40-PCE). The blank control group without deposition is referred to as PCE.

### 2.5. Hydrophobic Modification with Hexadecyltrimethoxysilane (HDTMS)

To further enhance the hydrophobic properties of the foams, surface modification was performed using hexadecyltrimethoxysilane (HDTMS).

The five groups of time-gradient samples after TEOS deposition (x-PCE), as well as the blank control group (0-PCE), were each divided into two portions, with one portion (groups 7–12) left untreated as a control.

The modification solution was prepared as follows: 105 g of anhydrous ethanol was mixed with 35 g of deionized water, and the pH of the mixture was adjusted to 4–5 using oxalic acid. Hexadecyltrimethoxysilane (1 g) was then added, and the solution was stirred until clear. The foam samples that required modification were immersed in this solution for 3 h, followed by drying at 60 °C to obtain hydrophobically modified composite foam materials.

The final products after HDTMS modification were designated as H-x-PCE, where H indicates the hydrophobic modification and x again corresponds to the prior TEOS deposition time (e.g., H-60-PCE).

### 2.6. Characterizations

The chemical composition, microstructure, wettability, and comprehensive performance of the prepared foams were systematically characterized using the following techniques. The microscopic morphology and pore structure of the foam samples were observed using a Hitachi SU 8200 cold field scanning electron microscope at a primary electron energy of 10.0 kV. Prior to observation, the samples were sputter-coated with a thin platinum layer using an MC1000 ion sputtering apparatus for 90 s at a current of 25 mA to enhance conductivity. The samples were cut open to expose their internal structure, and the SEM was used to observe the cross-sectional pore structure of the foam.

The pore size distribution and porosity of the samples were characterized by a Mercury intrusion porosimeter (Micromeritics-AutoPore IV 9500). The pore diameter d corresponding to each applied pressure P was calculated according to the Washburn equation:

(1) $$d=-\frac{4\gamma cos\theta }{P}$$

where *γ* is the surface tension of mercury (0.485 N·m^−1^) and *θ* is the contact angle between mercury and the polymer. The cumulative intrusion volume at each pressure step was converted into cumulative pore volume, and the differential intrusion volume was plotted as a function of pore diameter (with a logarithmic *x*-axis).

The total pore volume was taken as the maximum cumulative intrusion volume, and the apparent porosity was calculated as:

(2) $$Porosity=\frac{V_{pore}}{V_{bulk}}\times 100\%$$

where *V_pore_* is the total intruded pore volume and *V_bulk_* is the bulk volume of the sample. The median and average pore diameters reported in the manuscript are those calculated from the cumulative intrusion curve.

Chemical structures of the samples were characterized by FTIR spectroscopy to identify functional groups.

The surface wettability was evaluated by measuring the water contact angle on a Sindin SDC-200S contact angle measuring instrument. A deionized water droplet was gently dropped onto the freshly cut cross-section of the foam samples to evaluate the internal deposition time (e.g., H-60-PCE).

The compression performance was tested using an Instron 2367 electronic universal material tester at a speed of 2 mm/min. The specimens were prepared with a cross-sectional area of 20 mm × 20 mm and a gauge length (height) of 5 mm, ensuring consistent geometry for reliable comparison. Five parallel specimens were selected for each group to ensure statistical reliability. Among the obtained stress–strain curves, the specimen whose response was closest to the group average was chosen as the most representative sample for plotting and subsequent analysis.

The thermal stability of the samples was investigated using a thermogravimetric analyzer. Samples were heated from room temperature to 800 °C under a nitrogen atmosphere at a heating rate of 10 °C/min.

The absorption capacity for various oils and organic solvents was determined by immersing a pre-weighed dry foam sample into the liquid until saturation. The absorption capacity (Q, g/g) was calculated using the equation:

(3) $$k=\frac{m_{s}-m_{0}}{m_{0}}$$

where *m*_0_ and *m*_s_ are the masses of the sample before and after absorption, respectively. For the cyclic stability tests, the oil-saturated sample was placed in an ultra-filtration centrifuge tube and centrifuged to recover the absorbed oil, after which the sample was reused for the next cycle. The degradation behavior was evaluated by monitoring the mass loss of samples immersed in an aqueous buffer solution at 37 °C. At predetermined time intervals, the samples were removed, thoroughly rinsed with deionized water, dried to a constant weight, and then weighed to calculate the mass retention. The macroscopic morphological changes during degradation were documented.

## 3. Results and Discussion

### 3.1. Design of Experiment

**Figure 1:**
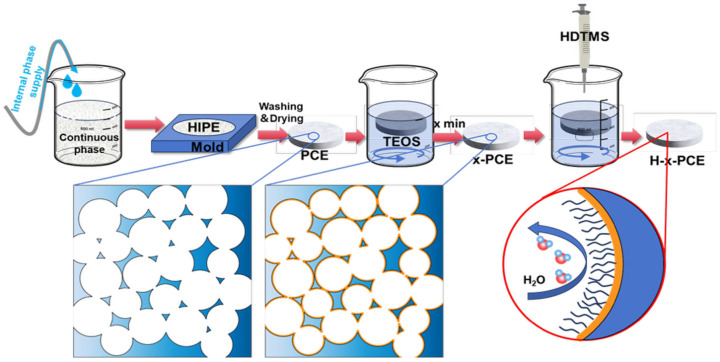
Schematic illustration of the fabrication process for the degradable superhydrophobic porous material (H-x-PCE).

A degradable and superhydrophobic material for oil–water separation was successfully fabricated through an integrated strategy, as illustrated in Figure 1. The process commenced with the construction of a porous polymeric scaffold using the high internal phase emulsion templating method. Specifically, a stable HIPE was formed by emulsifying an aqueous phase (internal phase, containing the initiator) into an oil phase (continuous phase, comprising vinyl-terminated PCL prepolymer, 2-ethylhexyl acrylate, toluene, etc.) [12,39,40]. This emulsion was then transferred into a designated mold and subjected to thermal polymerization. Subsequent washing and drying steps removed the template and unreacted monomers, yielding an interconnected porous polyester PCE foam.

To endow the material with superhydrophobicity, a two-step surface engineering approach was employed. The first step involved depositing silica particles onto the skeleton of the PCE via a sol–gel process [41]. The PCE samples were immersed in a precursor solution containing tetraethyl orthosilicate for varying durations (x minutes). Through hydrolysis and condensation reactions, a microscale rough layer was constructed on the polymer skeleton, resulting in samples x-PCE foams. The second step involved the hydrophobic modification of the silica-deposited foam using hexadecyltrimethoxysilane. The long alkyl chains of HDTMS were grafted onto the silanol groups of the x-PCE surface via chemical bonding, significantly reducing the surface energy [37,38]. The final superhydrophobic composite material is denoted as H-x-PCE. This design ingeniously integrates the degradability of the polymer, the high porosity and interconnected structure imparted by the HIPE template, the micro-roughness provided by silica, and the low surface energy introduced by HDTMS, laying a foundation for achieving efficient and environmentally benign oil–water separation.

### 3.2. Surface Morphology and Pore Structure Evolution

**Figure 2:**
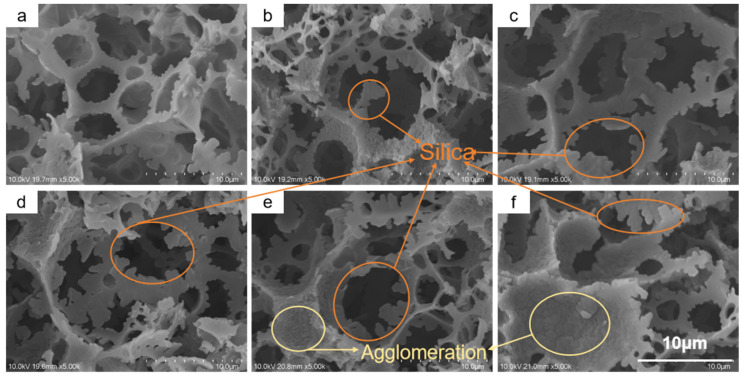
Scanning electron microscopy images of the pristine PCE and the H-x-PCE samples prepared with different TEOS deposition times. (**a**) PCE. (**b**) H-20-PCE. (**c**) H-40-PCE. (**d**) H-60-PCE. (**e**) H-80-PCE. (**f**) H-100-PCE.

SEM analysis clearly revealed the decisive influence of TEOS deposition time on the material’s microstructure. Figure 2a shows the pristine PCE foam without deposition, exhibiting a macroporous structure formed via the HIPE templating method. The pore struts are relatively smooth, and the interconnecting windows are clear and wide, providing an ideal pathway for rapid fluid transport.

After 20 min of TEOS deposition and HDTMS modification, the resulting H-20-PCE (Figure 2b) shows the initial formation of a micro-rough structure, with sparse silica particles beginning to appear on the skeleton surface. As the deposition time extends to 40 min (H-40-PCE, Figure 2c), the silica coverage increases markedly. The particles aggregate on the struts and begin to form fine, dendritic protrusions. It is noteworthy that the size of the interconnecting windows already shows a detectable reduction at this stage. When the deposition time reaches 60 min (H-60-PCE, Figure 2d), the polymer skeleton becomes so thoroughly enveloped by densely packed, dendritic silica structures that the underlying polymer framework is almost completely obscured. This complex structure composed of silica “dendrites” is crucial for trapping air and achieving superhydrophobicity. However, the continued deposition also alters the pore structure, as the windows become further narrowed by the thickening deposited layer.

At even longer deposition times (80 and 100 min, Figure 2e,f), the surface roughness continues to increase, but adverse effects become prominent. Excessive silica deposition not only thickens the pore struts but, more critically, leads to significant blockage of the interconnecting windows. In Figure 2e,f, many originally open windows can be observed being partially or completely “stitched” shut by the newly formed silica structures, a result of silica agglomeration [42]; sol–gel silica with abundant silanol groups is more hydrophilic and thus agglomerates more readily than hydrophobic silica [43]. While this pore closure might enhance the stability of the hydrophobic state, it is anticipated to be detrimental to the oil penetration rate and may compromise the material’s mechanical flexibility. Therefore, the TEOS deposition time needs to be optimized within a specific range to balance the acquisition of superhydrophobicity with the preservation of the porous structure.

**Figure 3:**
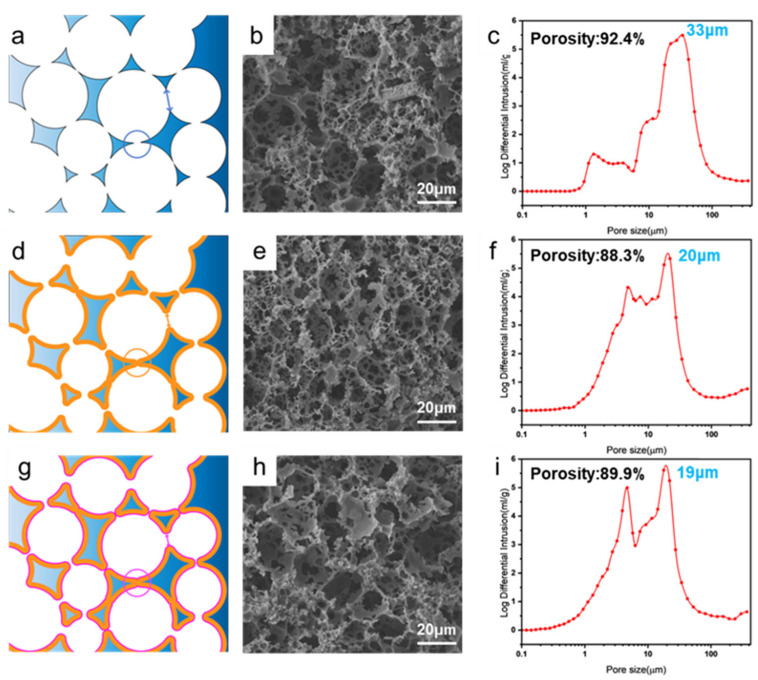
Pore structure characterization of PCE, 60-PCE, and H-60-PCE. (**a**) Two-dimensional schematic diagram of the pore structure for PCE. (**b**) Representative SEM image of PCE. (**c**) Pore size distribution curve from mercury intrusion porosimetry for PCE. (**d**) Two-dimensional schematic diagram of the pore structure for 60-PCE. (**e**) Representative SEM image of 60-PCE. (**f**) Pore size distribution curve from mercury intrusion porosimetry for 60-PCE. (**g**) Two-dimensional schematic diagram of the pore structure for H-60-PCE. (**h**) Representative SEM image of H-60-PCE. (**i**) Pore size distribution curve from mercury intrusion porosimetry for H-60-PCE.

Building upon the SEM observations, schematic illustrations and mercury intrusion porosimetry (MIP) were employed to quantitatively verify the effects of TEOS deposition and hydrophobic modification on the pore structure, as shown in Figure 3.

The schematic diagrams in Figure 3a,d,g visually demonstrate the structural evolution: from the original large pores in PCE, to the narrowed pores and partially filled small pores in 60-PCE due to silica deposition, resulting in an overall denser structure. The corresponding SEM images (Figure 3b,e,h) confirm this trend, showing narrowed interconnecting windows and an increased number of closed pores after the 60 min deposition.

MIP data provided quantitative support for the morphological observations. The pore size distribution curves in Figure 3c,f,i show a distinct decrease in both the peak pore size and the average pore size after silica deposition. Specifically, the peak pore size decreased slightly from approximately 33 μm for both 60-PCE and H-60-PCE. Concurrently, the porosity measured by MIP also exhibited sequential changes. The porosity of pristine PCE was 92.4%. After 60 min of TEOS deposition, the porosity of 60-PCE slightly decreased by 4.43 percentage points to 88.3%, directly attributed to the silica layer occupying part of the pore volume. Interestingly, after HDTMS modification, the porosity of H-60-PCE slightly increased to 89.9%. We speculate that this might be due to the hydrophobic modification significantly reducing the hydrophilicity of the polymer skeleton, thereby decreasing its water absorption capacity in the test environment and leading to an elevated apparent porosity.

In summary, while silica deposition successfully constructed a micro-rough structure, it inevitably led to pore size reduction and a slight decrease in porosity. Nevertheless, the final H-60-PCE material maintained a high level of porosity, which is crucial for its subsequent oil–water separation performance.

### 3.3. Mechanical Properties

**Figure 4:**
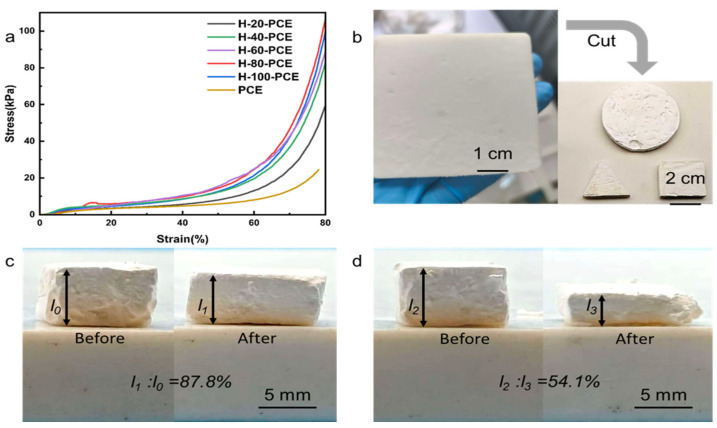
Mechanical properties of the porous foams. (**a**) Compressive stress–strain curves of PCE and H-60-PCE. (**b**) Optical photographs of PCE and H-60-PCE. (**c**) Recovery of PCE after being compressed to 20%. (**d**) Recovery of H-60-PCE after being compressed to 20%.

The mechanical strength of the material is crucial for its practical application and cyclic stability in oil–water separation processes. The compressive properties of PCE and H-60-PCE were compared, as shown in Figure 4.

The compressive stress–strain curve (Figure 4a) clearly shows that the compressive strength of H-60-PCE was significantly enhanced compared to the pure polymer matrix PCE after silica deposition and HDTMS modification. We attribute this primarily to the deposited silica particles forming a rigid, encasing SiO_2_ structure on the flexible polymer skeleton [44,45]. This structure effectively disperses and bears the applied stress, thereby markedly improving the material’s resistance to deformation.

For the mechanical performance at 80% strain, the average compressive strengths of the 100 min group, 60 min group, and pure PCE polymer matrix were 122.8 ± 7.1 kPa, 98.6 ± 8.3 kPa, and 24.6 ± 5.2 kPa, respectively. This corresponded to compressive strengths that were approximately five times higher for the 100 min group and approximately four times higher for the 60 min group compared with the pure PCE matrix compared to the pure PCE, with standard deviations indicating relatively consistent results within each group. These improvements further confirm that silica deposition and modification substantially enhance the mechanical strength and stability of the material.

However, this rigid SiO_2_ shell enhances strength at the cost of some elasticity. A comparison of the recovery behavior after compression (Figure 4c,d) visually demonstrates that pristine PCE exhibited excellent elasticity with a high recovery rate of 87.8%. In contrast, the recovery rate of H-60-PCE was significantly reduced to 54.1%. This indicates that the presence of the rigid silica network hinders the complete recovery of the polymer chains upon unloading, leading to increased permanent deformation.

In summary, the surface modification introduces a trade-off in mechanical properties: a significant enhancement in compressive strength is achieved at the expense of some recoverability. This characteristic suggests that the material is more suitable for separation scenarios requiring mechanical robustness without undergoing extreme deformation.

### 3.4. Surface Wettability

**Figure 5:**
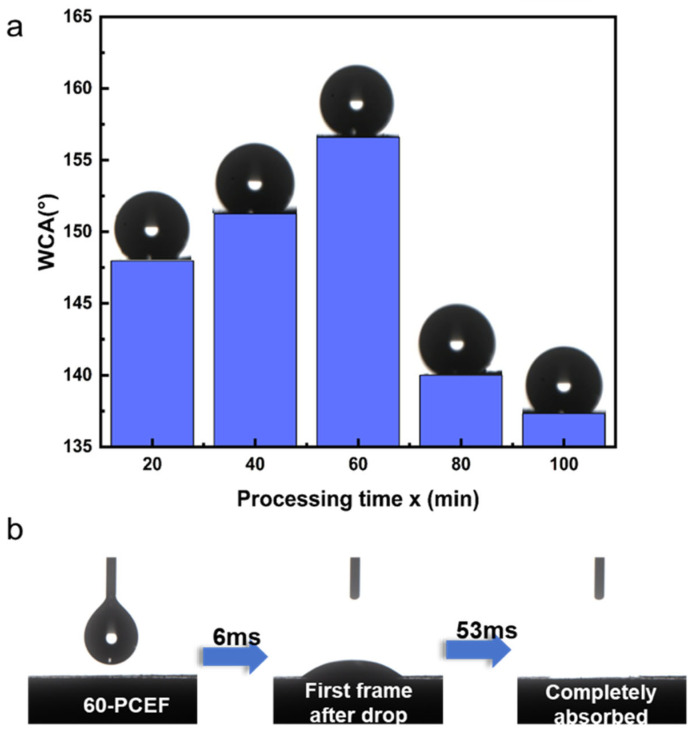
Wettability analysis of the porous foams. (**a**) Water contact angles measured on the internal surface of H-x-PCE samples prepared with different TEOS deposition times. (**b**) Sequence image of the water droplet penetration process on the internal surface of the 60-PCE sample.

We conducted water contact angle tests on the silica-based polymer with and without hydrophobic modification, as shown in Figure 5. To accurately evaluate the true wettability of the internal pore channels, all contact angle measurements were performed on freshly cut cross-sections of the foams to ensure reliability and representativeness.

As shown in Figure 5a, all H-x-PCE samples (only silica-deposited without HDTMS modification) exhibited typical hydrophobic characteristics. Figure 5b clearly demonstrates the behavior of a water droplet on the cross-section of 60-PCE: upon contact, the droplet was completely absorbed within 100 ms. This rapid, spontaneous wicking behavior originates from the abundant presence of hydrophilic silanol groups (-Si-OH) on the deposited silica surface, which attract water molecules via hydrogen bonding [46].

In contrast, after hydrophobic modification with HDTMS, all H-x-PCE samples achieved a transition from hydrophilic to hydrophobic. The water contact angles on their internal surfaces, as a function of TEOS deposition time, are plotted in Figure 5a. Notably, H-60-PCE demonstrated the optimal hydrophobic performance, with a contact angle as high as 156°, meeting the criterion for superhydrophobicity [18]. The mechanism for this transition lies in the replacement of the surface silanol groups by the long alkyl chains of HDTMS via chemical grafting, constructing a low-surface-energy layer. Combined with the microscale hierarchical roughness observed by SEM (Figure 2), according to the Cassie–Baxter model, air can be effectively trapped within this rough structure [47,48]. This causes the water droplet to primarily contact air and the low-surface-energy alkyl chains, ultimately leading to the superhydrophobic state. When the TEOS deposition time exceeds 60 min, the silica layer becomes excessively thick due to particle aggregation. This over-growth partially blocks the interconnecting pores and generates smoother local regions on the skeleton surface. These changes weaken the ability of the micro hierarchical structure to trap air pockets, reducing the stability of the Cassie–Baxter state and resulting in a slight decrease in the measured water contact angle.

In summary, wettability tests confirm the successful manipulation of the material’s wettability through surface chemical engineering. Silica deposition provides the platform for roughness construction and the modifiable hydroxyl groups, while HDTMS grafting is the key step for reducing the surface energy to a superhydrophobic level.

### 3.5. Chemical Composition and Thermal Stability Analysis

**Figure 6:**
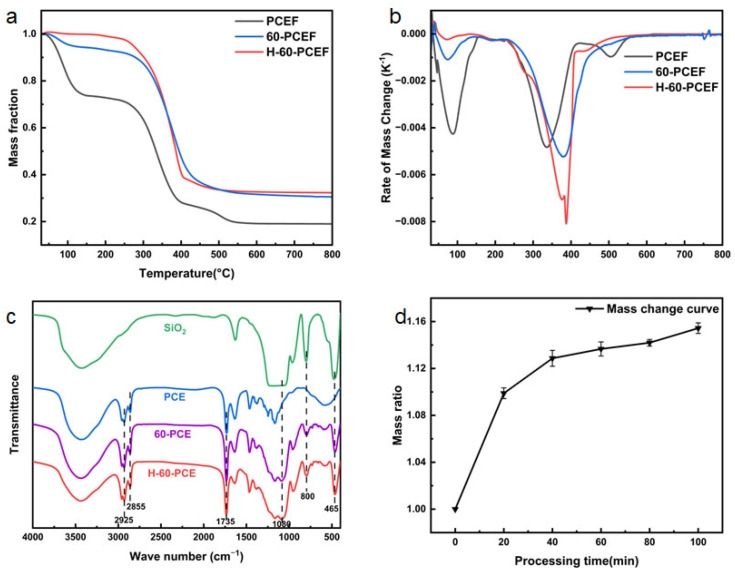
Chemical composition and thermal stability analysis of the materials. (**a**) Thermogravimetric analysis curves of PCE, 60-PCE, and H-60-PCE. (**b**) Derivative thermogravimetry curves of the corresponding samples. (**c**) Fourier-transform infrared spectra of PCE, 60-PCE, H-60-PCE, and pure silica. (**d**) The calculated mass increase due to silica deposition as a function of TEOS processing time.

To confirm the success of the surface modification and evaluate its impact on the material’s thermal stability, thermogravimetric analysis (TGA) and Fourier transform infrared (FTIR) spectroscopy were conducted, as shown in Figure 6. Additional thermal and structural characterization of the PCL precursors is provided in Figure S1, where the DSC curves of PCL-OH, PCL-TD, and the PCE foam reveal differences in melting behavior and crystallinity, and the FTIR spectra of PCL-OH and PCL-TD verify the successful vinyl end-group functionalization.

First, the thermal decomposition behavior was investigated by TGA. As shown in Figure 6a, the pristine PCE underwent its major mass loss between approximately 280 °C and 390 °C, corresponding to the breakdown and decomposition of the polymer backbone. After 60 min of TEOS deposition, 60-PCE exhibited a significantly higher residual mass at 800 °C than PCE. This directly proves the successful introduction of inorganic silica, which remains stable in this temperature range. The thermal decomposition behavior was further elucidated by the derivative thermogravimetry (DTG) curves (Figure 6b). A notable shift in the maximum decomposition temperature (T_max_) to a higher value was observed for both 60-PCE and H-60-PCE compared to the pristine PCE. This indicates that the deposited silica layer, even prior to HDTMS modification, enhances the thermal stability of the polymer composite to some extent. The silica network likely acts as a mass transport barrier, hindering the volatilization of decomposition products. This suggests that the grafted long alkyl chains act as a thermal barrier, slightly enhancing the maximum thermal stability temperature.

FTIR spectroscopy (Figure 6c) provides direct evidence for the surface chemical evolution. The spectrum of PCE showed the characteristic C = O stretching vibration of the ester group at 1735 cm^−1^. In the spectrum of 60-PCE, a broad and strong absorption band appeared around 1080 cm^−1^, assigned to the asymmetric stretching vibration of Si-O-Si, along with a Si-O bending vibration peak at 465 cm^−1^. These features, consistent with the spectrum of pure silica, reaffirm the presence of the silica coating. However, due to the strong background absorption from the C-H stretching vibrations of the polymer backbone itself in the 2800–3000 cm^−1^ region, the characteristic peaks of the grafted HDTMS alkyl chains could not be distinctly resolved in the H-60-PCE spectrum.

Based on the TGA data, the amount of silica deposition was quantified.

Let the initial mass of the pristine PCE foam be M. After TGA analysis up to 800 °C, its final residual mass fraction is y. For a modified sample, H-x-PCE, the initial mass is M + N, where N represents the mass of the deposited silica layer. Its final residual mass fraction is y. Assuming that the silica content remains stable at 800 °C while the polymer matrix decomposes, the mass conservation at this temperature gives:

(4) $$\left(M+N\right)\;z=M\;y+N$$

Solving Equation (2) for the silica mass fraction, *N*/(*M* + *N*), yields the final formula:

(5) $$\begin{array}{c} Silica\;Mass\;Fraction \end{array}=\frac{N}{M+N}=(\begin{array}{c} z-y \end{array})/(\begin{array}{c} 1-y \end{array})$$

where *y* is the residual mass fraction of PCE at 800 °C, z is the residual mass fraction of the H-x-PCE sample at 800 °C.

The calculated silica content data were plotted against x, the TEOS deposition time, as shown in Figure 6d. As plotted, the mass increase rate gradually rose with increasing TEOS processing time but plateaued between 60 and 100 min. This indicates that silica deposition proceeds rapidly in the initial stage and then approaches saturation due to reactant diffusion limitations or a reduction in active sites. This saturation trend corroborates the SEM observations where prolonged deposition led to pore structure blockage. The mass increase for 60-PCE compared to PCE was calculated to be approximately 13.7%.

Briefly speaking, the TGA and FTIR analyses jointly confirm the successful deposition of silica on the PCL-based foam skeleton and the subsequent hydrophobic modification via HDTMS. This process not only altered the surface chemistry from hydrophilic to superhydrophobic but also impacted its thermal stability. A brief overview of the phase state and thermal stability can be found in Figure S2.

### 3.6. Oil–Water Separation Performance

**Figure 7:**
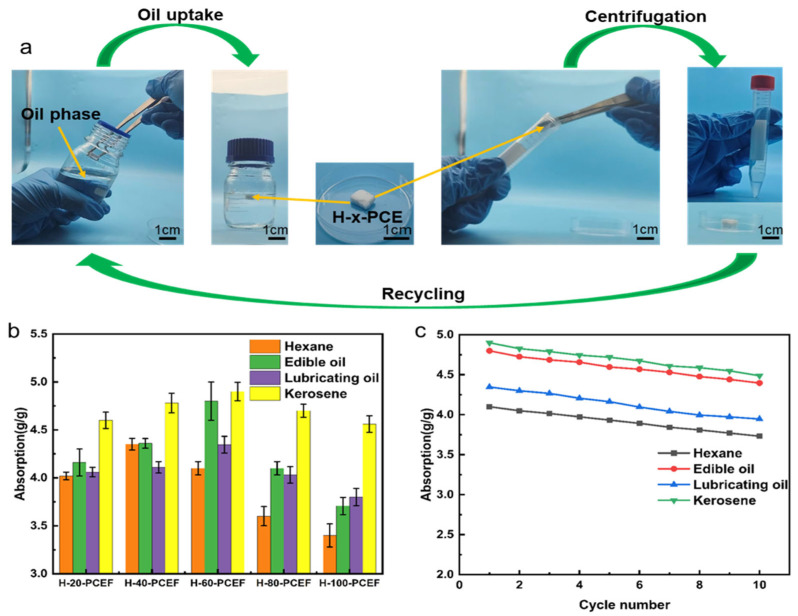
Oil–water separation performance of the porous foams. (**a**) Schematic diagram illustrating the selective oil absorption and cyclic regeneration process using H-x-PCE. (**b**) Absorption capacities of various H-x-PCE samples towards different oils/organic solvents. (**c**) Absorption capacity retention of H-60-PCE for different oils over 10 absorption–desorption cycles.

As the ultimate validation of the superhydrophobic porous material, the oil–water separation capability of the H-x-PCE foam series was systematically evaluated. The working principle is illustrated in Figure 7a: when the superhydrophobic H-x-PCE foam is placed at the oil–water interface, it completely repels the water phase while rapidly and selectively absorbing the oil phase via capillary forces. The oil-saturated material can be regenerated by centrifugation for oil recovery and material reuse, enabling cyclic operation. The data for different oils are provided in Table S1.

We first investigated the effect of TEOS deposition time on the absorption capacity. As shown in Figure 7b, all H-x-PCE samples exhibited high absorption capacities for the four tested oils/organic solvents (n-hexane, edible oil, lubricating oil, kerosene). We noticed that for low-viscosity n-hexane, the absorption capacity showed little variation with deposition time, being primarily governed by the total porosity of the material. For the higher-viscosity lubricating oil and edible oil, the absorption capacity initially slightly increased and then gradually decreased with longer deposition times. This is likely because the optimal micro-roughness constructed at moderate deposition times (e.g., 60 min) facilitates oil spreading and penetration, whereas the pore blockage caused by excessive deposition (see Figure 2e,f) increases the flow resistance, especially for viscous oils, within the channels, leading to reduced absorption capacity [49,50,51]. Overall, H-60-PCE demonstrated ideal and balanced absorption performance across all oils, consistent with its optimal superhydrophobicity and well-preserved porous structure identified in previous characterizations. The absorption capacities for n-hexane, edible oil, lubricating oil, and kerosene were 4.11, 4.79, 4.34, and 4.90 g/g, respectively. Although H-40-PCE exhibited a higher absorption capacity for n-hexane (4.36 g/g), H-60-PCE performed better overall across all oils. It is important to note that the observed differences in absorption capacities between various oils are largely attributed to the oils’ densities. When comparing the volumetric absorption capacities, the values closely resemble the foam’s porosity.

Cyclic stability is a crucial metric for practical application. The most performant sample, H-60-PCE, was subjected to 10 absorption–desorption regeneration cycles, with the results shown in Figure 7c. After 10 cycles, the material maintained a high absorption capacity retention for n-hexane, kerosene, and edible oil, indicating outstanding cyclic stability. H-60-PCE did not show structural collapse or failure of superhydrophobicity in any of the tests, which indicates that the designed structure meets the need of cyclic use.

In summary, H-60-PCE successfully integrates the flexibility of the degradable polymer matrix, the mechanical strength enhanced by the silica shell, and the superhydrophobicity imparted by surface modification, exhibiting efficient, highly selective, and recyclable oil–water separation capability.

### 3.7. Degradation Performance

**Figure 8:**
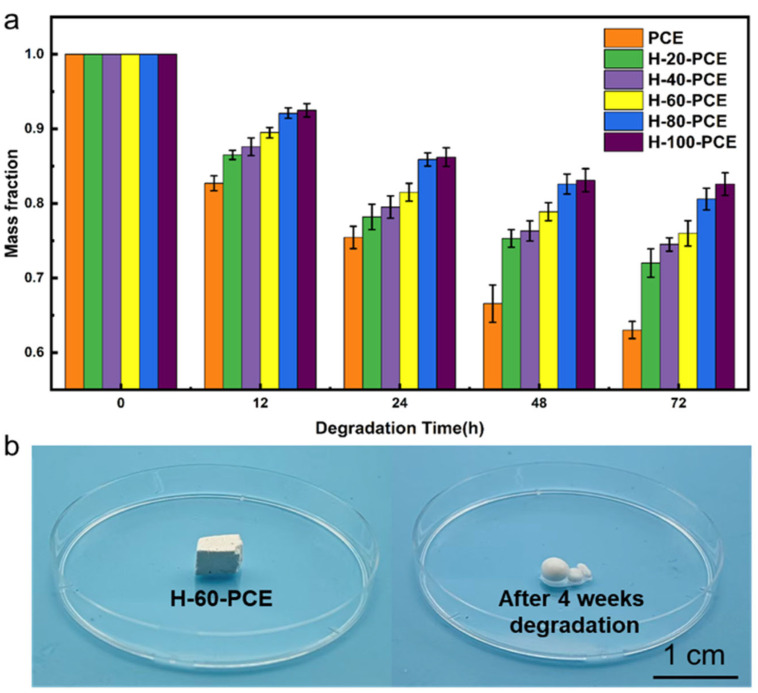
Degradation performance of the porous foams. (**a**) Mass retention of PCE and various H-x-PCE samples as a function of time in a degradation environment. (**b**) Macroscopic image of the H-60-PCE sample after 4 weeks of degradation.

The environmental sustainability of the material is a core innovation of this work. The degradation behavior of the PCE and H-x-PCE series was evaluated through hydrolysis tests under accelerated conditions (pH = 12), with the mass change monitored over time (Figure 8a).

As expected, the pristine PCE foam exhibited the most rapid degradation, with a mass loss of approximately 30% within 72 h, attributable to the hydrolysis of the ester linkages. In contrast, the degradation rates of all H-x-PCE samples were significantly retarded under the same conditions. For instance, H-60-PCE showed markedly higher mass retention than PCE after 72 h. This result unequivocally demonstrates that the conformal silica/HDTMS composite coating acts as a highly effective physical barrier [52,53]. This hydrophobic silica shell significantly impedes water penetration, thereby delaying the hydrolysis of the underlying PCL matrix and ensuring stability during its operational lifespan.

It is important to note that while the coating delays degradation, the material remains fundamentally degradable. Figure 8b shows the macroscopic structure of H-60-PCE after 4 weeks of hydrolysis, revealing obvious collapse and fragmentation, consistent with mass loss data exceeding 70% over one month. This confirms that the protective layer is ultimately compromised, allowing for extensive bulk degradation.

For practical end-of-life management, the used material can be mechanically pulverized. This process would disrupt the integral silica layer, exposing the degradable polymer interior directly to the environment [54]. Consequently, the degradation rate of the crushed material is expected to be significantly enhanced, potentially approaching that of the pristine PCE. This combination of service-life stability and triggered degradability at end-of-life presents a promising strategy for sustainable materials. Further investigation into the correlation between mechanical disruption and the subsequent degradation rate in natural environments represents a valuable direction for future research.

## 4. Conclusions

This work presents a materials-design strategy combining HIPE templating, copolymer network formation, and surface engineering to create a degradable porous foam for efficient oil–water separation. By integrating a PCL-based backbone with EHA-derived flexibility and crosslinking, the foam develops a two-level pore structure with large voids and window pores, enabling rapid fluid transport and high sorption capacity. Silica deposition via a TEOS-derived sol–gel process introduces microscale roughness, and HDTMS grafting stabilizes the Cassie–Baxter wetting state. These structural and chemical features result in a superhydrophobic, superoleophilic, and mechanically robust material with strong recyclability and selective absorption of oils across a broad viscosity range.

The foam exhibited a hierarchical pore structure, with porosity ranging from 92.4% to 88.3% after silica deposition and 89.9% after hydrophobic modification. The compressive strength improved significantly, with 122.8 kPa for the 100 min group compared to 24.6 kPa for the pure PCE matrix. Wettability analysis confirmed a maximum water contact angle of 156°, essential for oil–water separation. The foam also demonstrated excellent absorption capacities (4.11–4.90 g/g) and retained over 90% efficiency after 10 cycles. TG analysis confirmed the silica coating enhanced thermal stability, and the foam showed over 70% degradation within four weeks. These findings make the H-60-PCE foam a promising candidate for sustainable oil–water separation technologies.

Looking forward, conducting more systematic aging studies, including prolonged water immersion, extended sunlight exposure, salt-spray corrosion, and performance retention under high-humidity conditions, will be important for fully assessing the long-term outdoor durability of the foam in real application environments. Additionally, further investigations into the material’s performance in emulsified oil–water separation systems will be crucial to expand its applicability in more complex separation scenarios.

This study provides valuable insights and design principles that can guide the development of future degradable materials for oil–water separation. By exploring the integration of structural design, interfacial engineering, and polymer chemistry, the findings demonstrate how these elements can enhance both functional performance and environmental sustainability. This work serves as a solid foundation for further research on developing more efficient and sustainable separation technologies, with potential applications in environmental remediation and beyond, offering new opportunities for material innovations in addressing global challenges.

## Data Availability

The original contributions presented in this study are included in the article/Supplementary Materials. Further inquiries can be directed to the corresponding author.

## Supplementary Materials

The following supporting information can be downloaded at: https://www.mdpi.com/article/10.3390/polym17243254/s1, Figure S1. Thermal and structural characterization of PCL precursors. (a) DSC curves of PCL-OH, PCL-TD, and the PCE foam, showing differences in melting behavior and crystallinity. (b) FTIR spectra of PCL-OH and PCL-TD confirming successful vinyl end-group functionalization. Figure S2. Photographs of PCL-OH, PCL-TD, and PCE foam at room temperature (25 °C) and after heating at 65 °C. Table S1. Physical properties of the oils used in this study.
