# Supplementary material for: Degradable Polymer-Based Oil–Water Separation Materials Prepared by High Internal Phase Emulsion Templating Method and Silica-Modification
Source: Polymers (Basel). 2025 Dec 6;17(24):3254. doi: 10.3390/polym17243254 (PMC12737294; doi:10.3390/polym17243254)
Supplement: Supplementary file 1 [file polymers-17-03254-s001.zip › polymers-3989526-supplementary.pdf]

1. Added test for PCL and PCE foam

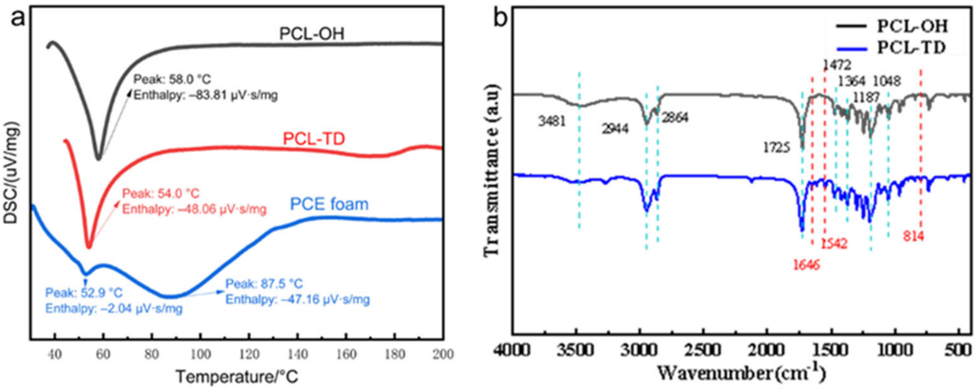

Figure S1. Thermal and structural characterization of PCL precursors. (a) DSC curves of PCL-OH, PCL-TD, and the PCE foam, showing differences in melting behavior and crystallinity. (b) FTIR spectra of PCL-OH and PCL-TD confirming successful vinyl end-group functionalization.

The DSC curves clearly show the thermal transitions associated with each precursor. PCL-OH exhibits a well-defined melting peak at 58.0 °C with a large enthalpy change (–83.81 µV·s/mg), consistent with its semicrystalline nature. After end-group vinylation, PCL-TD displays a melting peak at 54.0 °C with a significantly reduced enthalpy (–48.06 µV·s/mg), indicating a decrease in crystallinity due to incorporation of the reactive acrylate end-groups. This reduction of crystallinity is expected for chemically modified PCL and confirms the structural change prior to HIPE polymerization.

The FTIR spectra further verify the successful end-group functionalization. Both PCL-OH and PCL-TD retain the characteristic ester C=O stretching vibration near 1720 cm^{-1} and the C–O–C stretching bands typical of the PCL backbone. However, PCL-TD shows additional absorption features corresponding to vinyl functionality—specifically the C=C stretching vibration (~1635 cm^{-1}) and =C–H bending modes (~810–950 cm^{-1}). These peaks are absent in PCL-OH. Their appearance directly confirms the introduction of vinyl groups, which enables subsequent copolymerization with acrylate monomers in the HIPE system.

Because these DSC and FTIR data focus on confirming precursor modification rather than the performance of the final oil–water separation foams, we believe they fit best in the Supporting Information. This placement allows readers who are interested in precursor characterization to access the full details while keeping the main text focused on the development and performance of the HIPE-derived separation materials.

2. Thermal stability test explanation

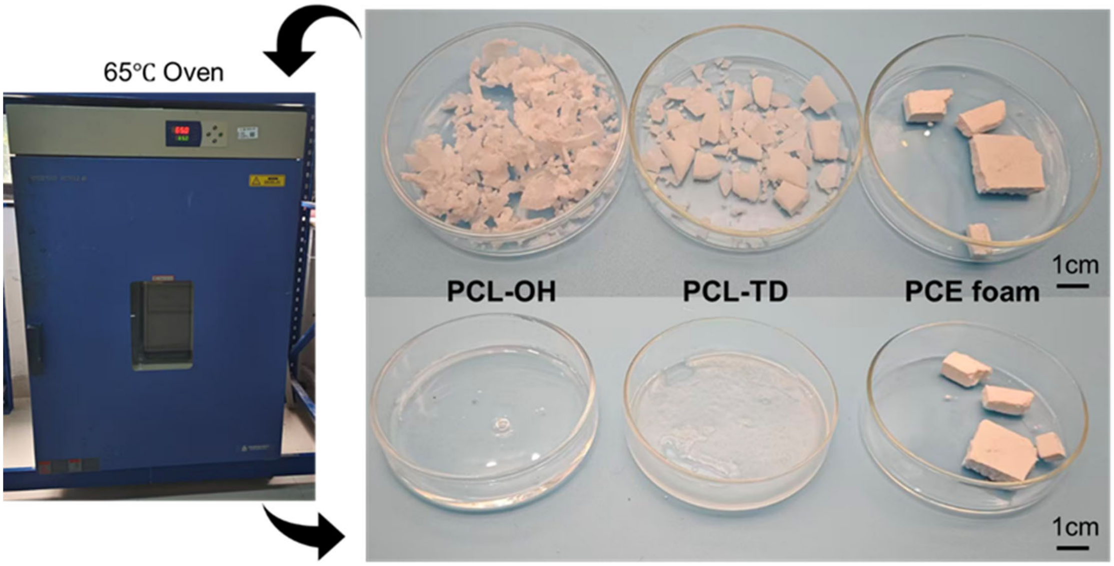

Figure S2. Photographs of PCL-OH, PCL-TD, and PCE foam at room temperature (25 °C) and after heating at 65 °C.

The vinyl-terminated PCL prepolymer is co-polymerized with 2-ethylhexyl acrylate (EHA) and EGDMA to form a chemically crosslinked PCL-co-EHA network. As a result, the polymer is no longer a semicrystalline thermoplastic but a crosslinked elastomeric network, which does not exhibit melting behavior at ~60 °C. Therefore, the shape stability of the foam is not compromised near the melting temperature of neat PCL.

Additionally, in Figure S1, we measured the low-temperature DSC for all three materials. The absorption peak area for the PCE foam was smaller than that of PCL-OH and PCL-TD, indicating a different thermal transition profile. Moreover, the TGA analysis (as presented in the manuscript) shows that the pyrolysis temperature for these

materials occurs between 280 °C and 390 °C, further confirming that, under normal working conditions, the thermal stability of the foam is excellent.

3. Data on oil products

The oil samples used in this study included n-hexane provided by Macklin Bio-chemical Technology, light fuel oil kerosene from Suzhou Shanjie New Material corpora-tion, industrial lubricating oil from Kunlun Lubricant, and common edible oil from Ara-wana. This variety of oil types aims to provide a wide range of oil chemistries and viscosi-ties.

Table S1. Physical properties of the oils used in this study.

**Oil sample**	**Typical compositio n / grade**	**Density ρ (g·cm⁻³)**	**Viscosity (dynamic μ, mPa·s or cP)**	**Kinematic ν (cSt)**
n-Hexane	Analytical solvent	0.66 g·cm⁻³ @ 20 °C	0.33 mPa·s	0.50 cSt
Kerosene	Petroleum kerosene	0.80 g·cm⁻³ @ 25 °C	1.64 mPa·s	2.0 cSt
Lubricatin g oil	ISO VG 68 hydraulic oil	0.88 g·cm⁻³ @ 25 °C	59.8mPa·s	68 cSt
Edible oil (Arawana)	Treated as soybean oil	0.917 g·cm⁻³ @ 25 °C	49.5 mPa·s	54 cSt
